# Complete mitochondrial genome sequences of two parasitic/commensal nemerteans, *Gononemertes parasita* and *Nemertopsis tetraclitophila* (Nemertea: Hoplonemertea)

**DOI:** 10.1186/1756-3305-7-273

**Published:** 2014-06-19

**Authors:** Wen-Yan Sun, Dong-Li Xu, Hai-Xia Chen, Wei Shi, Per Sundberg, Malin Strand, Shi-Chun Sun

**Affiliations:** 1 Institute of Evolution & Marine Biodiversity, Ocean University of China, 5 Yushan Road, Qingdao 266003, China; 2 Department of Biological and Environmental Sciences, University of Gothenburg, PO Box 463, SE-405 30 Gothenburg, Sweden; 3 Key Laboratory of Marine Bio-resource Sustainable Utilization (LMB), South China Sea Institute of Oceanology, Chinese Academy of Sciences, 164 West Xingang Road, Guangzhou 510301, China; 4 Swedish Species Information Centre, Swedish University of Agricultural Sciences, Box 7007, SE 75007 Uppsala, Sweden

**Keywords:** Nemertea, *Gononemertes parasita*, *Nemertopsis tetraclitophila*, Parasitic/Commensal, Mitochondrial genome, Phylogeny

## Abstract

**Background:**

Most nemerteans (phylum Nemertea) are free-living, but about 50 species are known to be firmly associated with other marine invertebrates. For example, *Gononemertes parasita* is associated with ascidians, and *Nemertopsis tetraclitophila* with barnacles. There are 12 complete or near-complete mitochondrial genome (mitogenome) sequences of nemerteans available in GenBank, but no mitogenomes of none free-living nemerteans have been determined so far. In the present paper complete mitogenomes of the above two parasitic/commensal nemerteans are reported.

**Methods:**

The complete mitochondrial genomes (mitogenome) of *G. parasita* and *N. tetraclitophila* were amplified by conventional and long PCR. Phylogenetic analyses of maximum likelihood (ML) and Bayesian inference (BI) were performed with both concatenated nucleotide and amino acid sequences.

**Results:**

Complete mitogenomes of *G. parasita* and *N. tetraclitophila* are 14742 bp and 14597 bp in size, respectively, which are within the range of published Hoplonemertea mitogenomes. Their gene orders are identical to that of published Hoplonemertea mitogenomes, but different from those of Palaeo- and Heteronemertea species. All the coding genes, as well as major non-coding regions (mNCRs), are AT rich, which is especially pronounced at the third codon position. The AT/GC skew pattern of the coding strand is the same among nemertean mitogenomes, but is variable in the mNCRs. Some slight differences are found between mitogenomes of the present species and other hoplonemerteans: in *G. parasita* the mNCR is biased toward T and C (contrary to other hoplonemerteans) and the *rrnS* gene has a unique 58-bp insertion at the 5′ end; in *N. tetraclitophila* the *nad3* gene starts with the ATT codon (ATG in other hoplonemerteans). Phylogenetic analyses of the nucleotide and amino acid datasets show early divergent positions of *G. parasita* and *N. tetraclitophila* within the analyzed Distromatonemertea species, and provide strong support for the close relationship between Hoplonemertea and Heteronemertea.

**Conclusion:**

Gene order is highly conserved within the order Monostilifera, particularly within the Distromatonemertea, and the special lifestyle of *G. parasita* and *N. tetraclitophila* does not bring significant variations to the overall structures of their mitogenomes in comparison with free-living hoplonemerteans.

## Background

The phylum Nemertea (ribbon worm) includes about 1280 named species [1]. Most of them are free-living in marine, freshwater and terrestrial habitats, but there are about 50 species reported to be associated with other animals; host organisms include poriferans, cnidarians, bivalves, echiurans, crustaceans, echinoderms and ascidians. The position of Nemertea among metazoans was traditionally considered to be close to the acoelomate Platyhelminthes, but comparative ultrastructure studies and molecular phylogenetic analyses during recent decades have supported it to be a member of the Lophotrochozoa [2-6]. The phylogenetic relationship of the phylum is still unsettled in parts, and conclusions may be dependent on different markers and analytical methods [7-9]. A recent analysis based on four nuclear and two mitochondrial loci further suggested that an expanded taxon sampling at family and generic level was required for getting a better understanding of nemertean affinities [9].

To date, there are 12 complete or near-complete nemertean mitogenome sequences available in GenBank. From these, we can infer some interesting patterns in terms of genome organization. For instance, Palaeonemertea and Heteronemertea bear larger mitogenomes than the more recently diverged hoplonemertean taxon Distromatonemertea. The gene arrangement within the phylum is not conserved, but generally stable within each of the three major groups (Palaeo-, Hetero- and Hoplonemertea). Nevertheless, a fuller understanding of the evolutionary patterns of nemertean mitogenome evolution requires denser taxon sampling, particularly of taxa that have adopted unusual lifestyles, such as *Malacobdella* and *Carcinonemertes*. In the present study, we determined the first complete mitogenome sequences of two parasitic/commensal nemerteans, *Gononemertes parasita* Bergendal, 1900 and *Nemertopsis tetraclitophila* Gibson, 1990, which taxonomically belong to Monostilifera (a group that contains most known symbiotic nemerteans). *G. parasita* lives in the branchial chamber of some ascidians in European waters [10], whereas *N. tetraclitophila* has been recorded from the mantle cavity of the balanomorph barnacle *Tetraclita squamosa* (Bruguiére, 1789) in Hong Kong, China [11]. Worms of both species seem to be firmly associated with a host, and possess some adaptive features that might be related to none free-living lifestyle, e.g., the greater number of gonads than most free-living monostiliferans; the absence of a proboscis apparatus (*G. parasita*) [11,12]. Mostly based on reproductive adaptations, Roe has argued that *G. parasita* and another *Nemertopis* species living in barnacles (*Nemertopis quadripunctata* (Quoy & Gaimard, 1833), which feeds on the eggs of the barnacles and possibly on the barnacles themselves) should be regarded as parasites [13]. However, the ecology, particularly the feeding biology, of *G. parasita* and *N. tetraclitophila* has not been well understood. Therefore, the two species are cautiously mentioned as “parasitic/commensal” in the present paper.

## Methods

### Specimens and DNA extraction

*Gononemertes parasita* was collected from the branchial chamber of the sea squirt *Ascidia obliqua* Alder, 1863 near Tjärnö, Sweden. *Nemertopsis tetraclitophila* was collected from the mantle cavity of the barnacle *Tetraclita squamosa* in Shenzhen, China. For either species, total DNA was extracted from a single specimen using the Genomic DNA Extraction Kit (OMEGA) following the manufacturer’s instructions and stored at −20°C.

### PCR amplification and sequencing

Small fragments such as *cox1*, *rrnS-rrnL*, *cob* and *cox3* were amplified with universal primers, and then specific primers were designed for the amplification of long fragments (Additional file 1: Table S1). All PCR reactions were carried out in a reaction volume of 25 μl containing 12.5 μl Premix Taq (LA version 2.0) (TaKaRa Clone Tech), 0.5 μl each primer, 0.5 μl DNA template and 11 μl distilled H_2_O. The PCR amplifications were performed under the following conditions: 4 min at 94°C, followed by 35 cycles of 30 s at 94°C, 30 s at 48–50°C (according to primers), 1–10 min (according to the length of products) at 72°C, followed by a 10 min elongation. The PCR products were separated by agarose gel electrophoresis and purified using DNA gel extraction kit (OMEGA). The purified PCR products were ligated into *pEASY*-T1 vector (Transgen, China) and sequenced by primer walking on an ABI 3730 Sequencer.

### Genome assembly and annotation

All the sequences were compared with other nemerteans to prevent contaminations from a host or bacteria. The obtained fragments of mitogenomes were assembled with Codoncode Aligner 5.0.1. Identification of protein-coding genes and rRNA genes was performed by BLAST searches (http://www.ncbi.nlm.nih.gov/BLAST) and by alignment to known hoplonemertean mitogenomes. Most tRNA genes were identified by tRNAscan-SE 1.21 [14], and additional tRNA genes were inferred with RNAfold [15]. The mitogenome was visualized using CGView [16]. The nucleotide composition and codon usage were calculated with DAMBE 5 [17]. Multiple alignments of genes were generated by Clustal X [18] with default settings and amino acid translation was carried out using MEGA 5.0 [19]. The full mitogenome sequences of *Gononemertes parasita* [KF572481] and *Nemertopsis tetraclitophila* [KF572482] were submitted to GenBank and compared with *Cephalothrix hongkongiensis* [NC_012821], *Cephalothrix* sp. [NC_014869], *Iwatanemertes piperata* [KF719984], *Lineus viridis* [NC_012889], *Lineus alborostratus* [NC_018356], *Nectonemertes* cf. *mirabilis* [NC_017874], *Amphiporus formidabilis* [KC710979], *Emplectonema gracile* [NC_016952], *Paranemertes* cf. *peregrina* [NC_014865], *Zygeupolia rubens* [NC_017877], *Prosadenoporus spectaculum* [KC710980] and *Nipponnemertes punctatula* [KC710981].

### Phylogenetic analysis

Phylogenetic analyses of the 14 available nemertean mitogenomes were carried out as follows: i) nucleotide-level analysis of protein-coding genes, with 3rd codon position removed; ii) nucleotide-level analysis of protein-coding genes, with 3rd codon position removed, rRNA and tRNA genes, iii) amino acid-level analysis of protein-coding genes. The saturation test was carried out based on the transition and transversion substitutions *vs.* the Tamura-Nei (TN93) distance of three codon positions by DAMBE 5 [17], and the third codon position which tended to be saturated (the transition and transversion substitution values do not increase as the genetic distance increase) was not used in phylogenetic analyses. The outgroups *Katharina tunicata* [NC_001636] and *Terebratulina retusa* [NC_000941] were selected based on their close relationships with Nemertea in previous studies [20,21]. All datasets were aligned with Clustal X with default settings [18]. Poorly aligned positions were excluded using Gblocks Version 0.91b [22] allowing less strict flanking positions and other default parameters. For nucleotide sequences MODELTEST [23] and MRMODELTEST [24], and for amino acid sequences ProtTest 2.4 [25] were used to select the best-fit substitution models (the model parameters were estimated when the concatenated nucleotides/amino acids were treated as a single partition). Based on the Akaike Information Criterion (AIC), the best-fit model for nucleotides was the GTR + I + G and for amino acid sequences was the MtRev + G + F. The ML analysis was performed with PHYML 3.0 program (http://www.atgc-montpellier.fr/phyml/) [26] with 100 bootstrap replicates. Bayesian inference was conducted using MrBayes version 3.1.2 [27]. Four Monte Carlo Markov chains (MCMC) were run for 1,000,000 generations, sampling every 100 generations. The first 2500 trees were omitted as burn-in. To ensure convergence, the run was not ended until the average standard deviation of split frequencies reached <0.01 and the PSRF values were close to 1 for all parameters. To investigate the contribution of different genes, the nucleotide data matrix containing the 1st and 2nd codon positions, rRNA and tRNA sequences was subjected to a heuristic parsimony analysis (i.e. hsearch addseq = random nreps = 1000 swap = TBR multrees = yes start = stepwise) in PAUP* 4.0 [28] and TreeRot.v3 [29] was used to calculate the partitioned Bremer support (PBS) values [30,31] of each gene partition on the tree nodes.

## Results and discussion

### Genome organization and base composition

**Figure 1:**
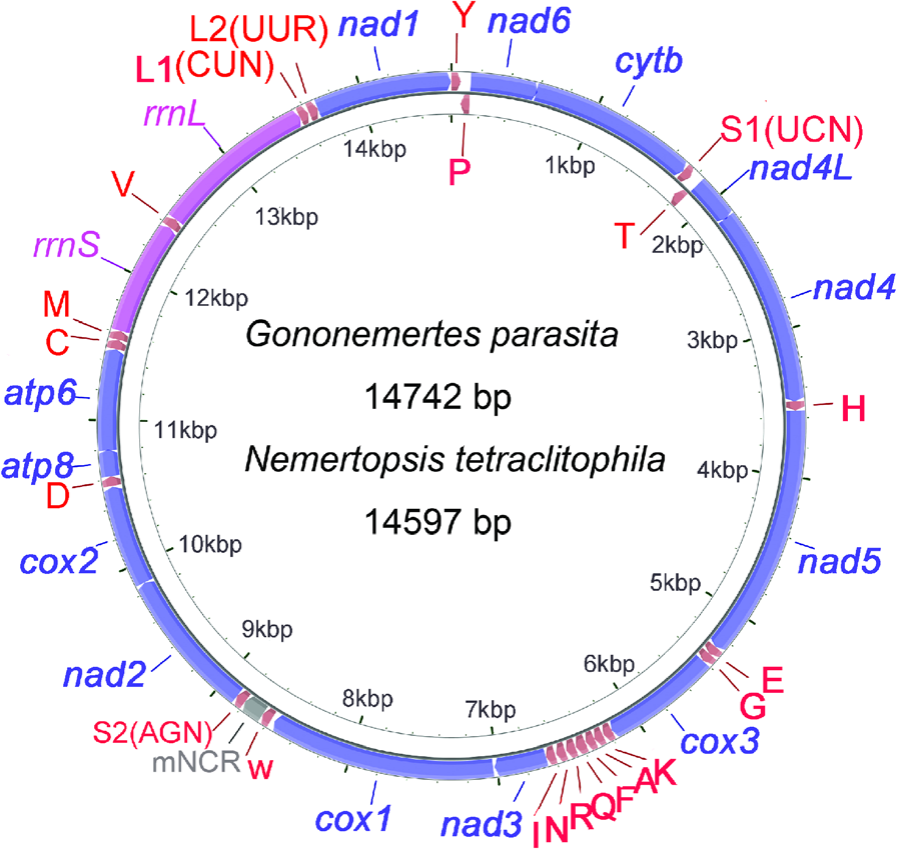
**Map of the mitochondrial genomes of *Gononemertes parasita *and *Nemertopsis tetraclitophila.*** Genes coded on the coding strand are arranged clockwise; those on the other strand are counter-clockwise. Thirteen protein-coding genes are shown in blue and two ribosomal RNA genes in pink. Transfer RNA genes are labeled by their single letter of corresponding amino acids. Major non-coding regions (mNCR) are represented in grey.

As observed in the previously determined Hoplonemertea mitogenomes, both of the present mitogenomes also include 13 protein-coding genes, two rRNAs and 22 tRNAs genes, all encoded on the coding strand except for *trnP* and *trnT* (Figure 1 and Table 1). The gene orders are identical to previously published hoplonemertean mitogenomes without exceptions. There are several overlaps throughout the two mitogenomes, for example, the 8-bp overlaps between *nad6* and *cob* (Table 1).

**Table 1:** **The mitochondrial genome organization of *Gononemertes parasita *** **and *Nemertopsis tetraclitophila***

**Genes**	***Gononemertes parasita***					***Nemertopsis tetraclitophila***				
	**From 5′ to 3′**	**Size (bp)**	**Start codon**	**Stop codon**	**3′ spacer**	**From 5′ to 3′**	**Size (bp)**	**Start codon**	**Stop codon**	**3′ spacer**
*trnY*	1-66	66			1	1-63	63			9
*trnP*^a^	133-68	66			3	138-73	66			8
*nad6*	137-604	468	ATG	TAG	−8	147-605	459	ATG	TAG	−8
*Cob*	597-1733	1137	ATG	TAA	5	598-1734	1137	ATG	TAG	−1
*trnS1*(UCN)	1739-1797	59			0	1734-1798	65			0
*trnT*^a^	1862-1798	65			4	1860-1799	62			2
*nad4L*	1867-2169	303	GTG	TAA	−7	1863-2165	303	ATG	TAG	−11
*nad4*	2163-3497	1335	ATG	TAG	23	2155-3504	1350	ATG	TAA	15
*trnH*	3521-3590	70			0	3520-3581	62			0
*nad5*	3591-5324	1734	GTG	TAG	−10	3582-5304	1723	ATG	T	0
*trnE*	5315-5381	67			6	5305-5368	64			2
*trnG*	5388-5451	64			2	5371-5434	64			1
*cox3*	5454-6233	780	ATG	TAG	7	5436-6215	780	ATG	TAG	5
*trnK*	6241-6309	69			0	6221-6281	61			−1
*trnA*	6310-6373	64			0	6281-6344	64			5
*trnF*	6374-6439	66			10	6350-6413	64			9
*trnQ*	6450-6518	69			0	6423-6493	71			3
*trnR*	6519-6583	65			8	6497-6560	64			2
*trnN*	6592-6657	66			7	6563-6626	64			0
*trnI*	6665-6729	65			1	6627-6690	64			7
*nad3*	6731-7084	354	ATG	TAA	1	6698-7040	343	ATT	T	0
*cox1*	7086-8621	1536	ATG	TAA	28	7041-8576	1536	ATG	TAG	33
*trnW*	8650-8718	69			0	8610-8676	67			0
mNCR^b^	8719-8838	120			0	8677-8813	137			0
*trnS2*(AGN)	8839-8905	67			−1	8814-8880	67			0
*nad2*	8905-9901	997	GTG	T	11	8881-9877	997	ATG	T	0
*cox2*	9913-10596	684	ATG	TAG	7	9878-10564	687	ATG	TAA	−2
*trnD*	10604-10668	65			0	10563-10629	67			0
*atp8*	10669-10839	171	GTG	TAG	7	10630-10797	168	GTG	TAG	5
*atp6*	10847-11539	693	ATG	TAG	6	10803-11489	687	ATG	TAG	−2
*trnC*	11546-11612	67			0	11488-11548	61			0
*trnM*	11613-11676	64			0	11549-11611	63			0
*rrnS*	11677-12513	837			0	11612-12384	773			0
*trnV*	12514-12578	65			0	12385-12450	66			0
*rrnL*	12579-13686	1108			0	12451-13540	1090			0
*trnL1*(CUN)	13687-13750	64			5	13541-13606	66			4
*trnL2*(UUR)	13756-13818	63			0	13611-13673	63			0
*nad1*	13819-14739	921	GTG	TAA	3	13674-14594	921	GTG	TAG	3

^a^the genes coded on the opposite strand.

^b^mNCR represents the major non-coding region.

**Figure 2:**
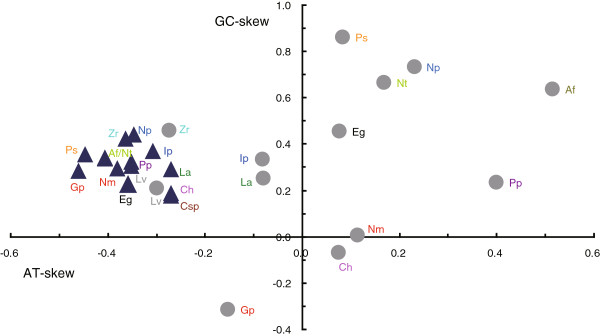
**Scatter plot of AT- and GC-skews in 14 nemertean species.** Values were calculated for the coding strand of the overall mitogenome sequences (▲) and the major non-coding region (*Cephalothrix* sp. not included because the major non-coding region of this species is incomplete) (●). AT-skew = (A-T)/(A + T); GC-skew = (G-C)/(G + C). Af = *Amphiporus formidabilis*, Ch = *Cephalothrix hongkongiensis*, Csp = *Cephalothrix* sp., Eg = *Emplectonema gracile*, Ip = *Iwatanemertes piperata*, Gp = *Gononemertes parasita*, Lv = *Lineus viridis*, La = *Lineus alborostratus*, Nt = *Nemertopsis tetraclitophila*, Np = *Nipponnemertes punctatula*, Nm = *Nectonemertes* cf. *mirabilis*, Ps = *Prosadenoporus spectaculum*, Pp = *Paranemertes* cf. *peregrina*, Zr = *Zygeupolia rubens*.

The nucleotide composition of the coding strand is biased toward T and A in these two mitogenomes, as is the case in most metazoan mitogenomes [32]. The A + T content of the coding strands in *G. parasita* and *N. tetraclitophila* is 68.8% and 71.2% respectively, which falls within the range of the previously sequenced nemertean mitogenomes (from 64.7% in *Lineus alborostratus* to 75.7% in *Cephalothrix* sp.) (Table 2). The A + T biased composition is particularly pronounced at the third codon position of the protein-coding genes (75.4% and 82.5%, respectively). The coding strands bear several poly-T stretches with the longest one being 20 Ts in *G. parasita* and 33 Ts in *N. tetraclitophila,* which have proved to be detrimental to PCR amplification [33,34]*.* Among lophotrochozoans, AT- and GC skews always show high inter- or intra-phylum variation, which might affect phylogenetic analyses [35]. The nucleotide skewness for the coding strands of *N. tetraclitophila* (AT-skew = −0.41, GC-skew = 0.34) and *G. parasita* (AT-skew = −0.46, GC-skew = 0.28) is biased toward T and G. A similar trend has been observed in other Nemertea mitogenomes (Figure 2): the negative AT-skew ranges from −0.46 (*G. parasita*) to −0.27 (*Cephalothrix* sp., *C. hognkongiensis* and *L. alborostratus*) and the GC-skew is always positive varying from 0.18 (*Cephalothrix* sp.) to 0.44 (*N. punctatula*). It is noteworthy that the nucleotide skews of the mNCRs are different among species (Figure 2), which reflects the relatively higher variability of mNCR. The mNCR of *G. parasita* is biased toward T and C, which is contrary to other hoplonemerteans.

**Table 2:** **Nucleotide compositions of *Gononemertes parasita *** **(Gp) and *Nemertopsis tetraclitophila *** **(Nt) mitogenomes**

**Feature**	**Length (bp)**		**A (%)**		**C (%)**		**G (%)**		**T (%)**		**A + T (%)**	
	**Gp**	**Nt**	**Gp**	**Nt**	**Gp**	**Nt**	**Gp**	**Nt**	**Gp**	**Nt**	**Gp**	**Nt**
Coding strand	14742	14597	18.6	21.1	11.2	9.5	20.0	19.3	50.3	50.0	68.8	71.2
Protein-coding genes^a^	11076	11058	15.8	18.1	11.5	9.6	20.4	19.6	52.3	52.8	68.1	70.9
1st codon position	3692	3686	18.0	20.2	12.6	11.5	25.2	25.0	44.3	43.2	62.2	63.5
2nd codon position	3692	3686	14.8	16.1	16.1	15.2	17.4	18.2	51.8	50.5	66.6	66.6
3rd codon position	3692	3686	14.6	17.9	6.0	2.1	18.6	15.4	60.8	64.6	75.4	82.5
tRNA genes	1445	1418	29.4	32.0	10.2	10.4	20.8	19.7	39.6	37.9	69.0	70.0
*rrnL* gene	1108	1090	26.7	28.6	9.8	8.9	17.5	17.6	45.9	45.0	72.7	73.6
*rrnS* gene	837	773	24.9	30.0	10.0	9.2	19.1	18.0	46.0	42.8	70.9	72.8
mNCR	120	137	30.0	43.1	19.2	4.4	10.0	21.9	40.8	30.7	70.8	73.7

^a^excluding stop codons.

### Protein-coding genes

**Figure 3:**
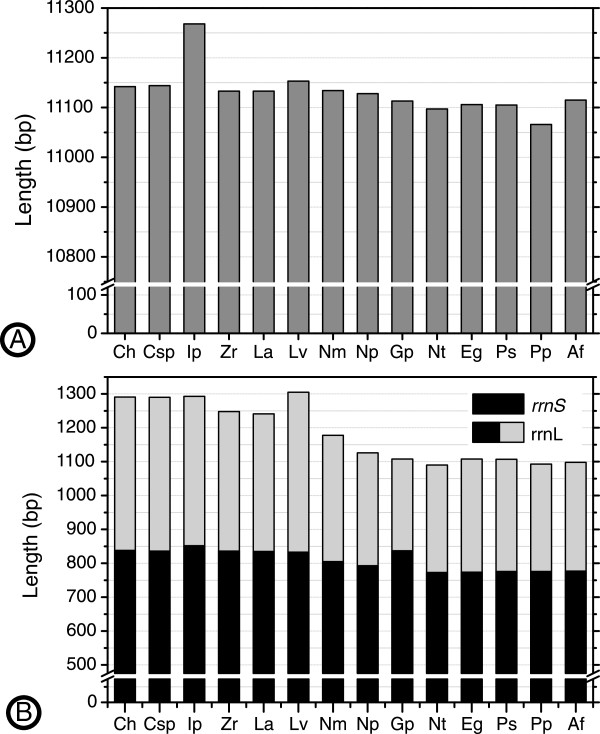
**Length comparisons of protein-coding genes (A) and ribosomal RNA genes (B) among 14 nemertean mitogenomes.** Abbreviations of species names see Figure 2.

The canonical start codons ATG and GTG are used in most protein-coding genes of the *G. parasita* and *N. tetraclitophila* mitogenomes. An exceptional case is the *nad3* gene of *N. tetraclitophila,* which was inferred to be initiated by the ATT codon (Table 1), and its length (343 bp) is shorter than that of other Monostilifera species (354 bp). Nonstandard initiation codons were also inferred in previously sequenced nemertean mitogenomes, e.g., the *cox1* (TCT) of *Cephalothrix* sp. and *C. hongkongiensis.* The majority of the protein-coding genes appear to use the stop codons TAA or TAG, except that the *nad5*, *nad3* and *nad2* genes in *N. tetraclitophila* and the *nad2* gene in *G. parasita* use a single T as the termination codon, most of which are adjacent to a protein-coding gene and occasionally a tRNA gene (Table 1). The incomplete termination codon T has been proposed to be converted into the complete stop codon TAA through polyadenylation during posttranscriptional mRNA processing [36]. The overall length of protein-coding genes in the known nemertean mitogenomes varies from 11066 to 11268 bp. The protein-coding genes in seven Monostilifera mitogenomes are shorter than that in the other nemerteans (Figure 3A). The two present mitogenomes do not exhibit apparent length change compared to other hoplonemertean mitogenomes, unlike in some parasitic insects whose protein-coding gene sizes are significantly smaller than those of free-living ones [37].

The overall nucleotide composition of 13 protein-coding genes in *G. parasita* and *N. tetraclitophila* mitogenomes are AT biased (68.1% and 70.9%, respectively). For both species, the third codon position has a considerably higher AT content (75.4% and 82.5%, respectively) than the first and second codon positions and the lowest content of C (Table 2). According to the analysis of relative synonymous codon usage (RSCU), the two- and four-fold degenerate codons prefer the one ending with T (Additional file 2: Table S2), for example, GCT (2.811) is more frequently used than the other three codons (0.297-0.486) for Ala. Corresponding to the high percentage of T in both mitogenomes, the most frequently used codon is TTT (17.6% and 16.3%, respectively), and Phe is the most frequently used amino acid (19.2% and 16.8%, respectively) (Additional file 2: Table S2). The other preferred amino acids in both species are Leu, Val, Gly and Ser, all of which might be associated with transmembrane functions. Similar codon usage and amino acid composition patterns have been observed in previously sequenced Nemertea mitogenomes [38].

### Ribosomal and transfer RNA genes

The ribosomal RNA genes (*rrnL* and *rrnS*) are located at the same location as in other nemertean mitogenomes, separated by *trnV*. The *rrnL* gene is 1,108 bp in *G. parasita* and 1,090 bp in *N. tetraclitophila*, and the A + T contents are 72.7% and 73.6%, respectively. The *rrnS* gene is 837 bp and 773 bp, and the A + T content is 70.9% and 72.8%, respectively (Table 2). At the 5′ end of *rrnS* gene in *G. parasita*, there is a region of 58 bp (TGTTTATTGGTATATTTTGATAAGTACTTTTAGTTTTATTCTATTTTTTTTCTTGTTT), which can neither be aligned with other nemertean *rrnS* sequences nor does it show any similarity with any remaining parts of the mitogenome, making the *rrnS* gene in *G. parasita* the longest among enoplan mitogenomes (Figure 3B). This insertion is also one major reason that *G. parasita* bears the largest mitogenome within Distromatonemertea. Except for *rrnS* of *G. parasita*, the rRNA genes of monostiferans are apparently shorter than that of other nemerteans (Figure 3B).

A + T contents in the tRNA genes is slightly lower than in the remainder of the mitogenomes. The anticodons of 22 tRNAs in both mitogenomes are the same as in other hoplonemerteans. All tRNA genes can be folded into conventional cloverleaf-like structures, except for *trnS1*(UCN) and *trnS2*(AGN) of *G. parasita,* and *trnS2*(AGN) of *N. tetraclitophila*. The structures of *trnS2* of both species conform to the secondary structure achieved for known hoplonemertean mitogenomes, all lacking a DHU-arm which is replaced by a DHU-loop [38,39]. *trnS1* of *G. parasita* was inferred to be 59 bp, which makes it one of the shortest known tRNA genes of nemerteans. It has a 5-T DHU-loop instead of a DHU-arm. Uncanonical secondary structures of tRNA genes occur frequently during animal evolution [40].

### Non-coding regions

There are a total of 265 bp and 250 bp non-coding nucleotides throughout the mitogenomes of *G. parasita* and *N. tetraclitophila*, accounting for 1.8% and 1.7% of the whole mitogenomes, respectively. The mNCRs are 120 bp and 137 bp, respectively, both located between *trnW* and *trnS2.* The A + T content (70.8% and 73.7%) of both mNCRs is slightly higher compared with the whole coding strands, but not as high as that of the third codon position (Table 2). Besides poly-T/C/G stretches, the two mNCRs have a similarity of 33%, which reflects a rapid evolutionary rate. Tandem repeats like those in *Amphiporus formidabilis* and *Nipponnemertes punctatula*[41] are not detected. In both, *N. tetraclitophila* and *G. parasita*, the mNCRs have the potential to fold into hairpin-like structures at the 5′ end (not shown), which might be involved in the beginning of replication and transcription [42]. The second longest mNCRs in the mitogenomes of *N. tetraclitophila* and *G. parasita* are both located between *cox1* and *trnW* (33 bp and 28 bp, respectively), in agreement with other Monostilifera species [41].

### Phylogenetic analysis

The concatenated datasets for amino acid and nucleotide sequences of the 13 protein-coding genes (excluding the 3rd codon position) yielded 3,056 and 6,721 aligned sites, respectively. The third dataset (comprising 8,962 nucleotide sites) was constructed by adding informative rRNA and tRNA gene sites to the above nucleotide dataset, which can help avoid directional migration resulting from only using protein-coding genes [43]. According to the Partitioned Bremer support (PBS) analysis [30], the rRNA and tRNA sequences contribute 17.7% and 11.7% (Table 3) of phylogenetic signal, respectively, making them promising for phylogenetic analysis.

**Table 3:** **Partitioned Bremer support values for each gene partition on the combined tree nodes in Figure**4**B**

**Gene**	**A**	**B**	**C**	**D**	**E**	**F**	**G**	**H**	**I**	**J**	**K**	**Total BS**	**BS contribution (%)**
1st codon position	181	86	1	13	43	16.5	−2	26	30	2	15	411.5	33.7
2nd codon position	141	86	30.3	6	99	2.5	7	41	26	12	1	451.8	36.9
rRNA	98	19	7.7	0	40	8.5	18	22	5	−5	3	216.2	17.7
tRNA	64	18	9	0	38	1.5	−7	7	3	5	5	143.5	11.7
Total	484	209	48	19	220	29	16	96	64	14	24		

The partitioned Bremer support values for each node add up to the total Bremer support (BS) values.

**Figure 4:**
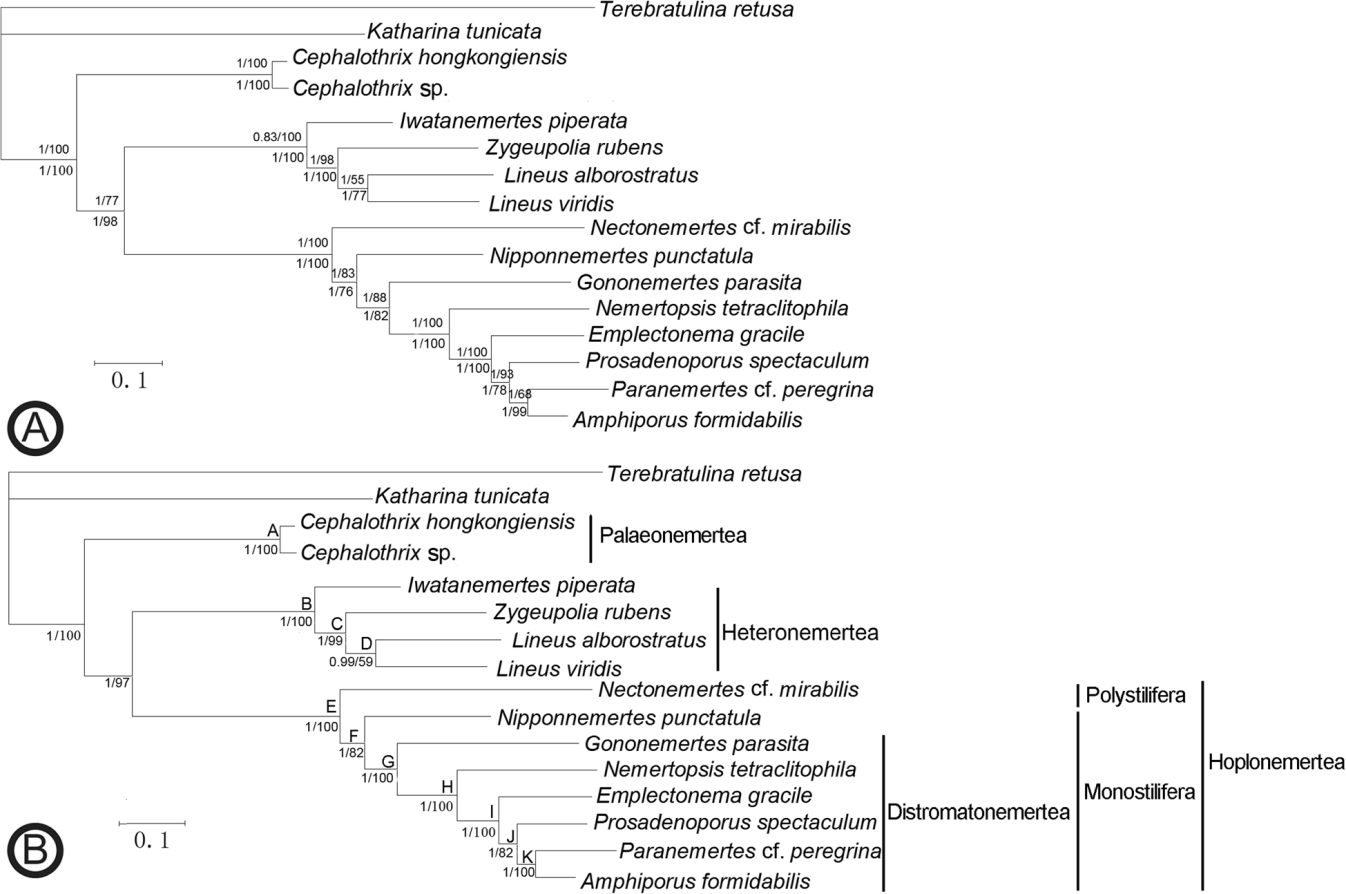
**Phylogenetic trees resulting from maximum likelihood and Bayesian inference. A**. Nucleotide sequences (3rd codon position removed)/amino acid sequences of 13 protein-coding genes (same tree topology obtained from the both datasets). **B**. Nucleotide sequences (3rd codon position removed) of protein-coding genes, rRNA and tRNA sequences. Numbers at the nodes correspond to posterior probabilities (left) and bootstrap proportions (right) (in tree **A**, the upper values are those of the nucleotide tree and the lower ones are those of the amino acid tree). Capital letters (A to K) in tree **B** correspond to the nodes for which Bremer support values were calculated (see Table 3).

Based on these three datasets, ML and BI analyses yielded identical tree topologies (Figure 4). All of them support the hypothesis that Hoplonemertea has a closer relationship with Heteronemertea than with Palaeonemertea, represented here by two *Cephalothrix* species that form the earliest divergent clade with high bootstrap values and posterior probabilities. As documented in previous studies [7,44], Polystilifera (*Nectonemertes* cf. *mirabilis*) is the sister group to Monostilifera; *Nipponnemertes* is sister to the other monostiliferans which make up the group Distromatonemertea [8]. The two present taxa, *G. parasita* and *N. tetraclitophila*, exhibit early divergent positions in the analyzed Distromatonemertea species. A recent analysis based on data of six genes also placed *G. parasita* in a basal Distromatonemertea clade containing mostly symbiotic and terrestrial species [9], whereas it was placed at a different position in the phylogenetic analysis of *cox1* and 18S rRNA sequences [45]. No similar species of the genus *Nemertopsis* have been studied in previous phylogenetic analyses. The position of the congeneric free-living species, *Nemertopsis bivittata*, was more or less different in previous analyses [8,9] and seems to be different from the placement of *N. tetraclitophila* in the present study, which calls for further studies about the interrelationships within the genus *Nemertopsis*.

## Conclusions

The complete mitochondrial genomes of *Gononemertes parasita* and *Nemertopsis tetraclitophila*, both of which possess some morphological characteristics adaptive to their lifestyle, are 14742 bp and 14597 bp, respectively. They are identical to the previously published mitogenomes of free-living hoplonemerteans in gene content and gene order, and have similar patterns in nucleotide richness and skewness. The length of whole genomes, as well as protein-coding genes and ribosomal RNA genes, is relatively conservative within Distromatonemertea and shorter (with the exception of the *rrnS* of *G. parasita*) than that of the other nemerteans. As in other hoplonemerteans, the coding strands of the present two mitogenomes bear some poly-T stretches; the tRNA genes usually exhibit cloverleaf-like structure except for *trnS*; the major non-coding regions exhibit AT-rich and hairpin-like structures that may be involved in transcription and replication. Some differences are found between the present mitogenomes and other hoplonemertean mitogenomes. For example, in *G. parasita* the mNCR is biased toward T and C (contrary to that in other hoplonemerteans) and the *rrnS* gene has a unique 58-bp insertion at 5′ end, and in *N. tetraclitophila* the *nad3* gene starts with the ATT codon (ATG in other hoplonemerteans). However, we cannot conclude that these differences are related to their special lifestyle, because similar variations may also exist among free-living nemerteans and available mitogenomic data of nemerteans are stilled limited. Phylogenetic analyses show that both *G. parasita* and *N. tetraclitophila* are early divergent within the analyzed Distromatonemertea species.

## Abbreviations

*atp6* and *atp8*: ATP synthase subunits 6 and 8; *cytb*: Cytochrome *b*; *cox1*-*3*: Cytochrome *c* oxidase subunits I-III; *nad1*-*6* and *nad4L*: NADH dehydrogenase subunits 1–6 and 4 L; *rrnL* and *rrnS*: The large and small subunits of ribosomal RNA; *trnX*: Transfer RNA molecules with the one-letter code of corresponding amino acid; DHU: Dihydrouridine; mNCR: Major non-coding region; PCR: Polymerase chain reaction; bp: Base pair.

## Competing interests

The authors declare that they have no competing interests.

## Authors’ contributions

S-CS conceived and designed the study. W-YS and D-LX performed the experiments. W-YS, D-LX and WS analyzed the data. MS, PS and S-CS collected and identified specimens. WY-S drafted the manuscript. S-C S, H-XC and PS revised the manuscript. All authors read and approved the final manuscript.

## Supplementary Material

Additional file 1: Table S1 PCR primers used to amplify the mitochondrial genomes of *Gononemertes parasita* and *Nemertopsis tetraclitophila.* Click here for file

Additional file 2: Table S2 Percentage of codon usage and relative synonymous codon usage (RSCU) of the 13 protein-coding genes in the mitogenomes of *Gononemertes parasita* (Gp) and *Nemertopsis tetraclitophila* (Nt). Click here for file
