# Supplementary material for: Complete mitochondrial genome sequences of two parasitic/commensal nemerteans, Gononemertes parasita and Nemertopsis tetraclitophila (Nemertea: Hoplonemertea)
Source: Parasit Vectors. 2014 Jun 19;7:273. doi: 10.1186/1756-3305-7-273 (PMC4081467; doi:10.1186/1756-3305-7-273)
Supplement: Additional file 1: Table S1 — PCR primers used to amplify the mitochondrial genomes of Gononemertes parasita and Nemertopsis tetraclitophila. [file 1756-3305-7-273-S1.doc]

# Additional files

**Additional file 1 PCR primers used to amplify the mitochondrial genomes of *Gononemertes parasita* and *Nemertopsis* *tetraclitophila***

| Gene/gene blocks and primer name | Sequence (5’→3’) | References |
| --- | --- | --- |
| Universal primers |  |  |
| *rrnS-rrnL* |  |  |
| rrnS-F | TGTGCCAGCTTCCGCGGTTATAC | Chen et al, 2011 |
| rrnL-R | CCGGTCTGAACTCAGATCACGT | Palumbi, 1996 |
| *cox1* |  |  |
| LCO-1490 | GGTCAACAAATCATAAAGATATTGG | Folmer et al, 1994 |
| HCO-2198 | TAAACTTCAGGGTGACCAAAAAATCA | Folmer et al, 1994 |
| *cox3* |  |  |
| cox3F | TGCGWTGAGGWATAATTTTATTTATT | Turbeville and Smith, 2007 |
| cox3R | ACCAAGCAGCTGCTTCAAAACCAAA | Turbeville and Smith, 2007 |
| *cob* |  |  |
| cytbF | GGWTAYGTWYTWCCWTGRGGWCARAT | Boore, 2000 |
| cytbR | GCRTAWGCRAAWARRAARTAYCAYTCWGG | Boore, 2000 |
| Newly designed primers |  |  |
| *Gononemertes parasita* |  |  |
| *cox1-rrns* |  |  |
| SW-COI-F | TGGCAGGAATCTCATCTAT | Present study |
| SW-12s-R | AATAACGGGGTGCCTAATC | Present study |
| *rrnL-cob* |  |  |
| SW-16s-F | CAGAGGGATAACAGCGTA | Present study |
| SW-cyb-R | GCGATGGAAACAATAACA | Present study |
| *cob-cox3* |  |  |
| SW-cyb-F | TTCTTTTGGTGATAGCGTT | Present study |
| SW-cox3-R | AAGCCCACTAACACGACT | Present study |
| *cox3-cox1* |  |  |
| SW-cox3-F | TATGAGGGATGGGGTATT | Present study |
| SW-COI-R | ATAGATGAGATTCCTGCCA | Present study |
| *Nemertopsis* *tetraclitophila* |  |  |
| *cox1-rrns* |  |  |
| B-COI-F | TGGTGCTGTGGAAAGTGG | Present study |
| B-12S-R | TGATTACGCTACCTTTGC | Present study |
| *rrnL-cob* |  |  |
| B-16S-F | TACCATAGGGATAACAGC | Present study |
| cytbR | GCRTAWGCRAAWARRAARTAYCAYTCWGG | Boore, 2000 |
| *cob-nad4* |  |  |
| B-CYB-F | GCGGTGGATAATGCTACT | Present study |
| Nnad4-R | ATAGAMCCWGAAACAGGAGC | Present study |
| *nad4-cox3* |  |  |
| B-nad4-F | GTTGCTCAGCGTTATCAT | Present study |
| B-cox3-R | ATGAGCCCAAGTAATCC | Present study |
| *cox3-cox1* |  |  |
| cox3F | TGCGWTGAGGWATAATTTTATTTATT | Turbeville and Smith, 2007 |
| cox1R | TAAACTTCAGGGTGACCAAAAAATCA | Folmer et al, 1994 |
