# Supplementary material for: Complete mitochondrial genome sequences of two parasitic/commensal nemerteans, Gononemertes parasita and Nemertopsis tetraclitophila (Nemertea: Hoplonemertea)
Source: Parasit Vectors. 2014 Jun 19;7:273. doi: 10.1186/1756-3305-7-273 (PMC4081467; doi:10.1186/1756-3305-7-273)
Supplement: Additional file 2: Table S2 — Percentage of codon usage and relative synonymous codon usage (RSCU) of the 13 protein-coding genes in the mitogenomes of Gononemertes parasita (Gp) and Nemertopsis tetraclitophila (Nt). [file 1756-3305-7-273-S2.doc]

**Additional file 2 Percentage of codon usage and relative synonymous codon usage (RSCU) of the 13 protein-coding genes in the mitogenomes of *Gononemertes parasita* (Gp) and *Nemertopsis* *tetraclitophila* (Nt).**

| Codon | Amino acid | Gp | |  | Nt | |
| --- | --- | --- | --- | --- | --- | --- |
|  |  | Frequency (%) | RSCU |  | Frequency (%) | RSCU |
| GCU | A | 2.82 | 2.811 |  | 3.09 | 3.328 |
| GCG | A | 0.41 | 0.405 |  | 0.16 | 0.175 |
| GCC | A | 0.30 | 0.297 |  | 0.14 | 0.146 |
| GCA | A | 0.49 | 0.486 |  | 0.33 | 0.350 |
| UGU | C | 1.63 | 1.905 |  | 1.79 | 1.886 |
| UGC | C | 0.08 | 0.095 |  | 0.11 | 0.114 |
| GAU | D | 1.68 | 1.550 |  | 2.25 | 1.976 |
| GAC | D | 0.49 | 0.450 |  | 0.03 | 0.024 |
| GAG | E | 1.84 | 1.766 |  | 1.22 | 1.059 |
| GAA | E | 0.24 | 0.234 |  | 1.09 | 0.941 |
| UUU | F | 17.61 | 1.839 |  | 16.30 | 1.936 |
| UUC | F | 1.54 | 0.161 |  | 0.54 | 0.064 |
| GGU | G | 3.30 | 1.749 |  | 3.83 | 2.097 |
| GGG | G | 2.90 | 1.534 |  | 1.41 | 0.773 |
| GGC | G | 0.46 | 0.244 |  | 0.16 | 0.089 |
| GGA | G | 0.89 | 0.473 |  | 1.90 | 1.041 |
| CAC | H | 0.41 | 0.429 |  | 0.19 | 0.197 |
| CAU | H | 1.49 | 1.571 |  | 1.74 | 1.803 |
| AUU | I | 5.04 | 1.888 |  | 5.86 | 1.946 |
| AUC | I | 0.30 | 0.112 |  | 0.16 | 0.054 |
| AAA | K | 0.73 | 0.982 |  | 0.87 | 1.049 |
| AAG | K | 0.76 | 1.018 |  | 0.79 | 0.951 |
| CUA | L | 0.54 | 0.462 |  | 0.11 | 0.120 |
| CUC | L | 0.11 | 0.092 |  | 0.05 | 0.060 |
| CUG | L | 0.46 | 0.393 |  | 0.16 | 0.180 |
| CUU | L | 3.58 | 3.052 |  | 3.28 | 3.639 |
| UUA | L | 5.55 | 1.057 |  | 6.48 | 1.135 |
| UUG | L | 4.96 | 0.943 |  | 4.94 | 0.865 |
| AUG | M | 1.16 | 0.843 |  | 1.63 | 1.043 |
| AUA | M | 1.60 | 1.157 |  | 1.49 | 0.957 |
| AAC | N | 0.19 | 0.173 |  | 0.05 | 0.043 |
| AAU | N | 2.00 | 1.827 |  | 2.50 | 1.957 |
| CCU | P | 1.73 | 2.327 |  | 2.28 | 3.170 |
| CCG | P | 0.22 | 0.291 |  | 0.24 | 0.340 |
| CCC | P | 0.35 | 0.473 |  | - | - |
| CCA | P | 0.68 | 0.909 |  | 0.35 | 0.491 |
| CAG | Q | 0.95 | 1.400 |  | 0.73 | 1.149 |
| CAA | Q | 0.41 | 0.600 |  | 0.54 | 0.851 |
| CGA | R | 0.24 | 0.581 |  | 0.27 | 0.588 |
| CGC | R | 0.11 | 0.258 |  | 0.05 | 0.118 |
| CGG | R | 0.49 | 1.161 |  | 0.38 | 0.824 |
| CGU | R | 0.84 | 2.000 |  | 1.14 | 2.471 |
| AGC | S | 0.22 | 0.168 |  | 0.08 | 0.063 |
| AGA | S | 0.68 | 0.526 |  | 1.36 | 1.047 |
| UCA | S | 0.38 | 0.295 |  | 0.52 | 0.398 |
| UCC | S | 0.30 | 0.232 |  | 0.14 | 0.105 |
| UCG | S | 0.46 | 0.358 |  | 0.19 | 0.147 |
| UCU | S | 5.44 | 4.232 |  | 5.21 | 4.021 |
| AGG | S | 0.89 | 0.695 |  | 0.76 | 0.586 |
| AGU | S | 1.92 | 1.495 |  | 2.12 | 1.634 |
| ACA | T | 0.49 | 0.783 |  | 0.30 | 0.473 |
| ACC | T | 0.11 | 0.174 |  | 0.03 | 0.043 |
| ACG | T | 0.08 | 0.130 |  | 0.19 | 0.301 |
| ACU | T | 1.81 | 2.913 |  | 2.01 | 3.183 |
| GUC | V | 0.35 | 0.151 |  | 0.11 | 0.046 |
| GUG | V | 1.03 | 0.441 |  | 1.11 | 0.471 |
| GUU | V | 6.93 | 2.968 |  | 7.30 | 3.092 |
| GUA | V | 1.03 | 0.441 |  | 0.92 | 0.391 |
| UGA | W | 0.68 | 0.500 |  | 1.38 | 0.962 |
| UGG | W | 2.03 | 1.500 |  | 1.49 | 1.038 |
| UAC | Y | 0.65 | 0.361 |  | 0.22 | 0.105 |
| UAU | Y | 2.95 | 1.639 |  | 3.93 | 1.895 |
